# Risk of Measles and Diphtheria Introduction and Transmission on Bonaire, Caribbean Netherlands, 2018

**DOI:** 10.4269/ajtmh.18-0824

**Published:** 2019-05-20

**Authors:** Regnerus A. Vos, Liesbeth Mollema, Jeroen Kerkhof, Johannes H. C. T. van den Kerkhof, Izzy Gerstenbluth, Alcira V. A. Janga-Jansen, Ymkje Stienstra, Hester E. de Melker, Fiona R. M. van der Klis

**Affiliations:** 1 Centre for Infectious Disease Control, National Institute for Public Health and the Environment, Bilthoven, The Netherlands;; 2 Department of Internal Medicine, University Medical Center/University of Groningen, Groningen, The Netherlands;; 3 Department of Epidemiology and Research, Medical and Health Service Curaçao, Willemstad, Curaçao;; 4 Department of Epidemiology, Curaçao Biomedical and Health Research Institute, Willemstad, Curaçao;; 5 Department of Public Health, Public Entity Bonaire, Kralendijk, Bonaire, The Netherlands

## Abstract

Endemic transmission of measles has been reestablished in Venezuela, and outbreaks of diphtheria remain ongoing across Latin America (LA). Hence, a large cross-sectional population-based serosurveillance study was conducted on Bonaire, one of the Dutch Leeward Antilles, to assess specific age and population groups at risk. Participants (aged 0–90 years) donated a blood sample and completed a questionnaire (*n* = 1,129). Antibodies against measles and diphtheria were tested using bead-based multiplex immunoassays. Our data revealed that immunity against measles is suboptimal, especially for those aged less than 5 years from Suriname, Aruba, and former Dutch Antilles (SADA), and adolescents from LA; and against diphtheria for persons aged more than 30 years, particularly among females and residents from SADA and LA. As refugees arrive persistently, health authorities on the Dutch Leeward Antilles should be on alert to detect early cases and prevent subsequent transmission. Ultimately, there is an urgent need for serosurveillance studies in the Caribbean region.

Whereas in 2016, the Americas was the first WHO Region to have reached measles elimination, endemic transmission has been reestablished in Venezuela as of August 2018.^1^ Concurrently, diphtheria is emerging rapidly as large outbreaks have been ongoing since mid-2016.^2^ Venezuela is facing a profound humanitarian crisis with the outflow of millions of its inhabitants into neighboring countries.^3^ Because of political developments and socioeconomic depression, the country faces lack of funding for public health activities. Together with shortages of supply of medicine, including vaccines, this resulted in a disrupted national immunization program (NIP).^4^ As of August 2018, 8,544 confirmed measles cases had been reported across the country, resulting in 62 deaths, and 1,992 suspected diphtheria cases, with 168 deaths.^1,2^ The massive outflow of unvaccinated and possibly infected Venezuelans to surrounding countries cause a substantial risk of introduction of vaccine-preventable diseases (VPDs).^3^ Neighboring countries in Latin America (LA) have already reported imported and autochthonous measles and diphtheria cases (e.g., Brazil [measles] and Colombia [both]), and corresponding deaths.^1,2^

The Dutch Leeward Antilles Aruba, Bonaire, and Curaçao are located in the southern Caribbean Sea nearby the northern coast of Venezuela. More than 25,000 Venezuelan refugees have arrived on these islands and this number is growing.^3^ Hence, considering the small size and limited capacity of these Antilles, large numbers of arrivals—which account for ∼10% of the total combined population—have great impact on the community and could potentially introduce measles and diphtheria in a population with possible susceptible pockets.

Vaccination is a highly effective method of preventing measles and diphtheria. On the Dutch Leeward Antilles, monovalent measles vaccination (one dose) for children aged 15 months was introduced in 1977 and was replaced by the measles–mumps–rubella (MMR) vaccine in 1988 for infants aged 14 months. A booster for 9-year-olds followed in 1991.^5^ Diphtheria-containing vaccines have been administered from the 1940s. The present NIP^5^ recommends five doses of diphtheria-tetanus-acellular pertussis–inactivated poliovirus vaccine (DTaP-IPV, at the ages of 2, 3, 4, and 11 months, and 4 years) and one dose of diphtheria-tetanus–inactivated poliovirus vaccine (DT-IPV) (at 9 years). On Bonaire, the early childhood vaccination coverage is 90% (at the age of 2 years); however, the coverage is below 70% at the age of 10 years. Fortunately, no cases of measles or diphtheria have been reported in the last decade.^6^

Supported by our cross-sectional population-based serosurveillance study (“Health Study Caribbean Netherlands,” for a brief description^7^) conducted on Bonaire in mid-2017, we present the population seroprevalence underpinning the potential emerging risk of measles and diphtheria introduction and transmission and discuss the corresponding preventive measures.

The study proposal was approved by the Medical Ethics Committee Noord-Holland, the Netherlands (METC-number: M015-022), and informed consent was obtained from all adult participants and parents or legal guardians of minors included in the study. From the population registry (*n* = 19,203), an age-stratified sample of 4,798 inhabitants (with age strata 0–11, 12–17, 18–34, 35–59, and 60–90 years) was drawn, of which *n* = 1,197 responded (net response rate: 26%). At the clinic, participants were requested to donate a fingerstick blood sample—which was collected via the dried blood spot method—and to complete a questionnaire on infectious diseases and other health-related factors (*n* = 1,129). Samples were air-shipped to the laboratory of the National Institute for Health and the Environment (RIVM), Bilthoven, the Netherlands, directly after the fieldwork period. IgG antibodies against measles and diphtheria were analyzed using bead-based multiplex immunoassays, as described previously.^8,9^ For measles, IgG antibody levels ≥ 0.120 international units per mL (IU/mL) were considered seropositive,^10^ and for diphtheria, 0.01 IU/mL was considered the minimum protective level.^11^

In this study, among those eligible for the NIP (i.e., until 41 and 64 years for measles and diphtheria, respectively), the vaccination registry showed that 463 participants (68.9%) received at least one dose of a measles-containing vaccine (more specifically, one dose: 248 [36.9%]; two or more doses: 215 [32.0%]) and 530 (55.8%) participants had been administered at least once with a diphtheria-containing vaccine (more precisely, one dose: 39 [4.1%]; two to five doses: 313 [32.9%]; six or more doses: 178 [18.7%]). From NIP-eligible participants without vaccination registry, 164 (78.5%) self-reported to have (partly) joined the NIP and 304 (73.1%) self-reported to have been administered with a diphtheria-containing vaccine as a child. The vaccination coverage (i.e., at least one dose based on registry or self-reporting) for measles was 93.4%, 93.9%, and 86.9% in age groups 0–11, 12–17, and 18–34 years, respectively, and for diphtheria, the vaccination coverage was 99.6%, 92.8%, 83.1%, and 74.4% in age groups 0–11, 12–17, 18–34, and 35–59 years, respectively.

Population-based estimates showed that the overall measles seroprevalence was 93.7% (95% CI: 91.9–95.4) with an overall geometric mean concentration (GMC) of 0.918 IU/mL (95% CI: 0.829–1.016). None of the infants aged less than 1 year in our sample (*n* = 15; all unvaccinated) had (maternal) antibodies above the cutoff (Figure 1). The seroprevalence for the age group 1–4 years—that is, after the first dose of the MMR vaccine at 14 months—was 85.2% (95% CI: 76.6–93.9) and steadily increased to 94.5% (95% CI: 90.8–98.3) for the age group 10–14 years, which most probably reflects the vaccine response after the (second) dose (mostly) administered at 9 years, also demonstrated by a slightly elevated GMC. Thereafter, the seroprevalence was below 95%—a level considered necessary for herd immunity^12^—until the age group 40–44 years. After the first MMR vaccination, GMCs remain above the protective cutoff and steeply increased from the age group 40–44 years onward, reflecting the people naturally exposed to the virus. The overall seropositivity was lowest for people from LA (91.0%), especially at the age group 12–17 years (64.0% [95% CI: 45.6–82.4] [data not shown]), and from Suriname, Aruba, and former Dutch Antilles (SADA) (93.8%) (Table 1), particularly at the age group 1–4 years (86.2% [95% CI: 77.1–95.2]) (data not shown).

**Figure 1.**
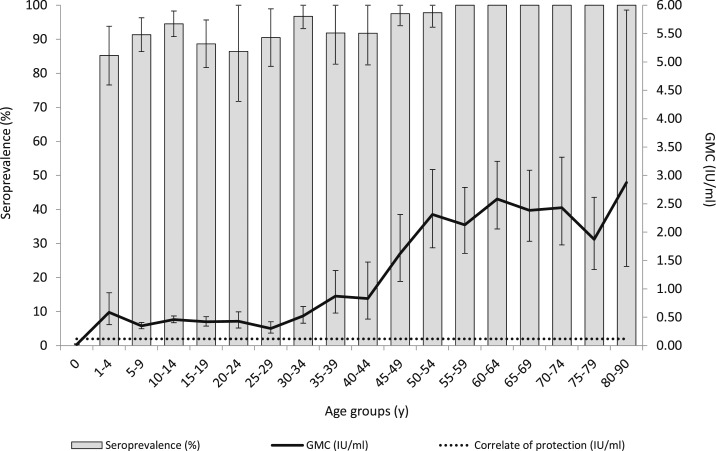
Weighted age-specific seroprevalence and geometric mean concentration (GMC) (with 95% CIs) of measles IgG antibodies in the general population of Bonaire, Caribbean Netherlands, 2017. Note: Antibody concentration ≥ 0.120 IU/mL was considered protective, that is, the correlate of protection. A seroprevalence of 95% is considered necessary for herd immunity.

**Table 1:** Weighted age-specific seroprevalence (with 95% CIs) of measles and diphtheria IgG antibodies in the general population of Bonaire, Caribbean Netherlands, 2017, by gender and age stratum, and ethnicity and age stratum

	No. (%) 1,129	Measles seroprevalence (95% CI)	Diphtheria seroprevalence (95% CI)
Gender and age stratum (years)			
Males	506 (44.8%)	93.4% (90.7–96.0)	82.7% (78.2–87.2)
0–11	141	83.6% (76.9–90.2)	99.1% (97.4–100.0)
12–17	85	87.9% (80.3–95.5)	95.5% (90.9–100.0)
18–34	51	90.2% (81.8–98.7)	83.9% (73.1–94.8)
35–59	96	96.5% (92.5–100.0)	76.7% (67.8–85.5)
60–90	133	100.0% (100.0–100.0)	77.0% (69.6–84.3)
Females	623 (55.2%)	94.0% (91.9–96.0)	73.5% (69.5–77.6)
0–11	130	83.6% (76.5–90.7)	97.6% (94.8–100.0)
12–17	96	93.8% (88.9–98.7)	94.6% (89.7–99.5)
18–34	109	92.8% (87.9–97.7)	86.0% (79.5–92.5)
35–59	146	95.6% (92.1–99.1)	57.5% (49.3–65.6)
60–90	142	100.0% (100.0–100.0)	65.4% (57.2–73.7)
Ethnicity* and age stratum (years)			
Indigenous Dutch and other Western countries†	143 (14.2%)	95.9% (92.1–99.7)	86.0% (79.5–92.4)
0–11	22	96.7% (90.4–100.0)	100.0% (100.0–100.0)
12–17	9	100.0% (100.0–100.0)	75.0% (45.1–100.0)
18–34	14	83.7% (62.8–100.0)	86.4% (68.8–100.0)
35–59	44	95.9% (90.4–100.0)	86.7% (75.3–96.1)
60–90	54	100.0% (100.0–100.0)	83.2% (73.4–93.0)
Suriname, Aruba, and former Dutch Antilles‡	803 (64.5%)	93.8% (91.7–95.8)	78.1% (74.3–81.9)
0–11	236	83.2% (77.9–88.5)	98.5% (96.7–100.0)
12–17	142	96.3% (93.0–99.5)	98.2% (96.0–100.0)
18–34	110	93.5% (88.1–98.8)	86.2% (78.3–94.1)
35–59	128	96.1% (92.4–99.7)	60.0% (51.1–69.0)
60–90	187	100.0% (100.0–100.0)	68.3% (61.2–75.3)
Latin America and other non-Western countries§	182 (21.3%)	91.8% (87.2–96.4)	73.5% (66.1–80.9)
0–11	13	68.5% (42.6–94.5)	93.9% (82.2–100.0)
12–17	30	65.4% (47.5–83.3)	87.4% (75.2–99.6)
18–34	36	88.2% (76.0–100.0)	81.1% (67.8–94.5)
35–59	69	96.1% (90.8–100.0)	68.1% (56.7–79.4)
60–90	34	100.0% (100.0–100.0)	68.6% (52.9–84.3)

* Ethnicity was unknown for one male in the age group 35–59 years.

† *n* = 41 (29%) participants from Western countries other than indigenous Dutch.

‡ Former Dutch Antilles includes the islands Bonaire, Curaçao, Saba, St. Eustatius, and St. Maarten.

§ *n* = 171 (94%) participants from Latin American countries within the group Latin America and other non-Western countries.

For diphtheria, 78.3% (95% CI: 75.2–81.3) of the overall antibody levels was above the minimum protective level (of 0.01 IU/mL), with a GMC of 0.047 IU/mL (95% CI: 0.042–0.053). After the last DT-IPV vaccine administered at the age of 9 years, the GMC rapidly declined and remained just above the minimum protective level from the age of 30 years onward. From 30 years onward, the overall seropositivity was below 75%—a level considered important for herd protection in adults^13^—namely, 69.3% (95%: CI: 65.0–73.7), aside from the age group 60–64 years (82.1%) (Figure 2). Notably, the seropositivity for females was significantly lower than that for males (73.5% versus 82.7%; *P* < 0.005) (Table 1), particularly at the age group 50–54 years (45.6% versus 88.0%; *P* < 0.001) (data not shown). Males slightly more often self-reported to be vaccinated because of their profession, a trip abroad, or military service in the past (29.6% versus 24.6%). Furthermore, the seropositivity was lowest among residents from SADA (78.1%) (Table 1), LA (72.9% [95% CI: 65.1–80.7]), and other non-Western countries (79.2% [95% CI: 56.7–100.0]) (data not shown)—who all self-reported to be less vaccinated than indigenous Dutch and people from other Western countries—especially at the age group 35–59 years (SADA: 60.0% [Table 1]; and LA: 67.2% [95% CI: 55.2–79.3] [data not shown]).

**Figure 2.**
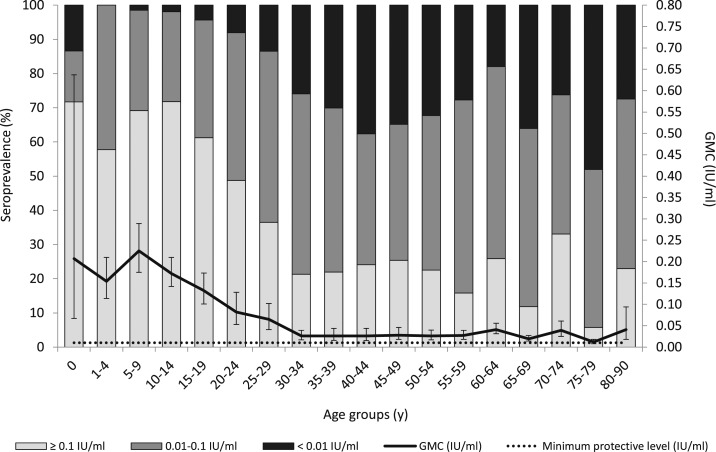
Weighted age-specific seroprevalence and geometric mean concentration (GMC) (with 95% CIs) of diphtheria IgG antibodies in the general population of Bonaire, Caribbean Netherlands, 2017. Note: Antibody concentration below 0.01 IU/mL was considered non-protective, 0.01–0.1 IU/mL provides basic protection (i.e., 0.01 IU/mL is the minimum protective level), and ≥ 0.1 IU/mL provides full protection. A seroprevalence of 75% is considered necessary for herd immunity in adults.

This is the first ever conducted serosurveillance study on Bonaire, providing important data demonstrating that immunity against measles and diphtheria is insufficient. As outbreaks of these VPDs are ongoing in surrounding countries and Venezuelan refugees are arriving constantly, the risk of introduction and subsequent transmission is present. The overall seroprevalence for measles was high (93.7%), however, not reaching the level considered necessary for herd immunity (i.e., 95%).^12^ Subgroups with the lowest seroprevalence are those aged less than 44 years, more specifically adolescents (aged 12–17 years) from LA and, most strikingly, infants aged less than 5 years from SADA. To decrease susceptibility in this vulnerable group and in line with countries in the region, the public health department on Bonaire has lowered the age for the second MMR vaccine from 9 years to 18 months as of January 1, 2019. Furthermore, the diphtheria overall seroprevalence (i.e., proportion of people with a minimum protective level) was rather low (78.3%). Waning immunity, indicated by declining GMCs with age, has been reported previously by others,^14–17^ and because of the increased exposure, this could be a potential risk on Bonaire. Risk groups include people aged more than 30 years, especially females and people from SADA and LA. Importantly, because measles and diphtheria are highly contagious, the probability of introduction and transmission is more likely when individuals who lack protection cluster together, for example, children at schools or people from the same cultural background or religion.^18^

Taken together, there is an urgent need for increased awareness on Bonaire, one of the Dutch Leeward Antilles, considering potential introduction of measles and diphtheria cases amid groups with lower seroprotection. Surrounding islands facing an ongoing influx of refugees should be on the alert too. The vaccination status of refugees remains to be verified on arrival if possible, with vaccinations offered to those who are eligible to ensure full protection. (Re)vaccination of risk groups who lack protection and people who are in close contact with refugees should be considered. In addition, early detection, rapid treatment, and well-coordinated source- and contact tracing (according to ring principle) are of great importance to prevent transmission and disease. Health-care workers, who should be well vaccinated themselves, must be aware of the control measures according to applicable guidelines. Diphtheria can cause severe complications (e.g., myocarditis), and the case fatality rate without treatment is 50%^19^; hence, a rapid supply of antitoxins (and antibiotics) should be facilitated. This, together with confirmation by laboratory diagnostics and notification of cases, is essential to control subsequent transmission. Last, serosurveillance studies in the Caribbean region are scarce. The present study enables us to carry out representative epidemiological (sub)analyses as we chose a robust design to diminish selective response, for example, instead of using residual sera, and weighted our sample on a set of sociodemographic factors to correctly represent the population of Bonaire. Ultimately, there is a need for data across the region to detect gaps in terms of population immunity and to further decrease the risk of imported and autochthonous transmission of VPDs. Preventive measurements as described here should be considered across the region in the meantime.
